# Performance of PICS bags under extreme conditions in the sahel zone of Niger

**DOI:** 10.1016/j.jspr.2018.01.007

**Published:** 2018-03

**Authors:** Ibrahim B. Baoua, Ousmane Bakoye, Laouali Amadou, Larry L. Murdock, Dieudonne Baributsa

**Affiliations:** a Université Dan Dicko DanKoulodo de Maradi, BP 465 Maradi, Niger; b Institut National de la Recherche Agronomique du Niger (INRAN), BP 240 Maradi, Niger; c Purdue University, West Lafayette, IN 47906, United States

**Keywords:** Grain storage, PICS bags, Sun exposure, Sahel, Niger

## Abstract

Experiments in Niger assessed whether extreme environmental conditions including sunlight exposure affect the performance of triple-layer PICS bags in protecting cowpea grain against bruchids. Sets of PICS bags and woven polypropylene bags as controls containing 50 kg of naturally infested cowpea grain were held in the laboratory or outside with sun exposure for four and one-half months. PICS bags held either inside or outside exhibited no significant increase in insect damage and no loss in weight after 4.5 months of storage compared to the initial values. By contrast, woven bags stored inside or outside side by side with PICS bags showed several-fold increases in insects present in or on the grain and significant losses in grain weight. Grain stored inside in PICS bags showed no reduction in germination versus the initial value but there was a small but significant drop in germination of grain in PICS bags held outside (7.6%). Germination rates dropped substantially more in grain stored in woven bags inside (16.1%) and still more in woven bags stored outside (60%). PICS bags held inside and outside retained their ability to maintain internal reduced levels of oxygen and elevated levels of carbon dioxide. Exposure to extreme environmental conditions degraded the external polypropylene outer layer of the PICS triple-layer bag. Even so, the internal layers of polyethylene were more slowly degraded. The effects of exposure to sunlight, temperature and humidity variation within the sealed bags are described.

## Highlights

• PICS bags exposed to the sun and high temperatures for few months maintain grain quality.
• Exposure to extreme environmental conditions for a few months minimally affected the performance of PICS bags.
• The longevity and reuse of PICS bags are reduced when stored outside for several months.

## 1 Introduction

In West Africa, cowpea (*Vigna unguiculata* Walp) production was estimated to be 4.5 million tons (FAO STAT, 2014). This essential food legume for rural populations is ravaged by insects in the field and during storage after harvest. Losses caused by bruchids are estimated to range from 25 to 95% after 3 to 4 months of storage (Singh et al., 1985). Over the last ten years, post-harvest protection of cowpea grain in the Sahelian zone of Africa has improved considerably thanks to the introduction and dissemination of the hermetic triple-bagging technology called PICS (Purdue Improved Crop Storage) (Baributsa et al., 2010). A PICS bag consists of a woven polypropylene outer bag and two internal polyethylene liners and costs about 2.0–3.0 USD in local markets in the region. The bags are effective in preserving grain quality for at least fourteen different crops. The technology has been directly demonstrated to more than 5 million farmers in 56,000 villages in Africa and at least 10 million bags have been sold by June 2017 (PICS, 2017).

Storage space is a major challenge in many Sub-Saharan African households. Some farmers store their grain-filled PICS bags under a tarpaulin outside but within their household compound. Others store PICS bags inside warehouses such that the bags are exposed to direct sunlight. In addition, the authors have received many inquiries from potential large-scale users asking about the effect of environmental conditions on PICS bags and their effectiveness when kept outside for several days or even weeks. Such large-scale users include development agencies that source food aid from local markers or private processing companies that buy grain from farmers but may not have available warehouse in rural areas to store large amounts of grain. Based on some of these field observations, we have recommended that users not expose their PICS bags to the sunlight in order to avoid the deterioration of the woven bags. The aim is to avoid any potential effect of environmental conditions such as temperature on grain quality. Even so, the question of grain quality in PICS bags stored outside under extreme weather conditions has never been systematically investigated.

The present study was conducted to better understand how environmental conditions affect the performance of hermetic storage systems such as PICS bags when the grain-filled bags are stored outside exposed to full sunlight. Given that storage space is sometimes a constraint in many communities in sub-Saharan Africa, the results of this present study will lead to more scientifically-based recommendations for the more efficient use of the technology.

## 2 Material and methods

Experiments were carried out at the INRAN Entomology laboratory in Maradi, Niger over a period of two seasons (November 26, 2015 to April 7, 2016): (1) The cold dry season extends from November 2015 to January 2016 with a monthly sunshine duration of 203–258 h. The daily average minimum temperature varied between 11.4 and 12.3 °C while the average maximum daily temperature was 30.0–38.0 °C; (2) the warm dry season from March 2016 to May 2016 which has a monthly sunshine duration of 221–288 h, a daily average minimum temperature ranging between 12.4 and 22.1 °C and a maximum daily temperature of 40.5–44.6 °C. (These data were collected from the website http://www.infoclimat.fr/climatologie/annee/2016/maradi/valeurs/61080.html).

The PICS 50 kg triple-bags used in these experiments were manufactured by Lela Agro (Kano, Nigeria). Cowpea grain (1200 kg) was purchased from a local market in Maradi. This grain, already naturally infested with cowpea bruchids, was thoroughly mixed to obtain a homogenous initial infestation. Woven bags and PICS bags in twelve replicates were each filled with 50 kg of the infested grain. Six filled PICS bags and six filled woven bags were stored outside exposed to the sun, and side by side on a raised platform inside a screen cage to avoid damage by animals. The other sets (six filled PICS bags and six filled woven bags) were stored on pallets inside the laboratory.

Data loggers, model EL-USB-2 (Lascar, Whiteparish, Wiltshire, Great Britain), were placed in one bag of each treatment to record temperature and relative humidity over the course of the experiments. Another data logger was placed outside to measure the ambient environmental conditions. Oxygen and carbon dioxide levels were monitored using a Mocon PAC Check Model 325 Headspace analyzer (Mocon, Minneapolis, MN, USA) fitted with a 20-gauge hypodermic needle for sampling through rubber septa or through the walls of the storage bags. We sealed punctures in bag walls with patches of electrician's tape. Oxygen and carbon dioxide data was collected from the second day of the experiment (November 27, 2015) until March 18, 2016 at which point some PICS bags had begun losing their airtightness.

The bags for each treatment were monitored daily to detect any changes in the physical condition of the outer woven polypropylene bags as well as the outer and inner polyethylene liners. This assessment was initially made to the exposed outer polyethylene bag. Next, its surface was touched lightly with a finger. Any crack, breakage or crumbling of the material was judged as degradation. Over time, cracking, crumbling and attendant falling away of the outer woven bag exposed the outer polyethylene bag to the sun and the elements. When this polyethylene layer became exposed it was likewise inspected and touched to determine its physical integrity. The process was repeated when the innermost polyethylene bag had become exposed after the middle bag had degraded and fallen away. In conjunction with inspections of physical integrity, the oxygen and carbon dioxide levels in the bag were determined with the Mocon device, as described above.

The initial bruchid infestation level in the cowpea grain was assessed at the beginning of the experiment by randomly collecting 64 samples of 500 g each. Each 500 g sample was sieved to separate and count dead and live adults. Pupae were counted as adults. Three random samples of 100 seeds were picked from each 500 g sample and soaked in water for 15 min to soften the grains. The water-imbibed seeds were cracked and opened to count the number of living and dead or desiccated larvae, pupae, and adults. At the end of the experiment, 18 samples of 500 g and 54 samples of 100 grains per each treatment were evaluated for dead and live larvae, pupae, and adults.

Data collected was recorded in Excel for calculating mean and standard errors. For temperatures and relative humidity, daily averages were determined as well as the correlations between the treatments and prevailing environmental conditions (temperature and relative humidity). Statistical analysis was done with SPSS software (Statistical Package for the Social Sciences), produced by IBM SPSS, Inc. in Chicago, Illinois. Analysis of Variance (ANOVA) followed by Least Significant Difference (LSD) tests was used to compare parameters related to oxygen and carbon dioxide, infestations and damage per treatments.

## 3 Results

**Fig. 1:**
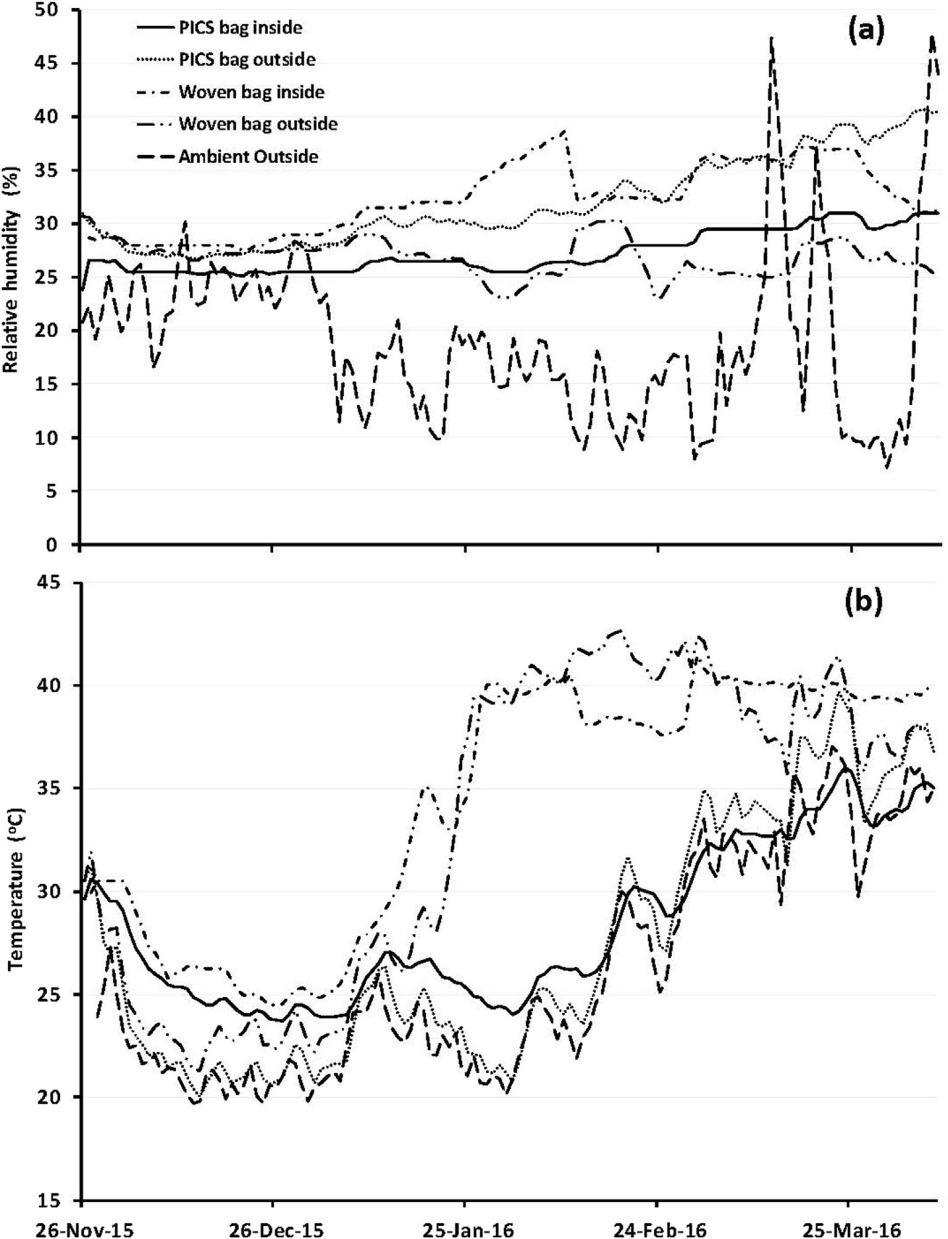
Average daily relative humidity (a) and temperature (b) in PICS triple bags and woven bags filled with naturally infested cowpea and stored at the INRAN Maradi Station in Niger for 4.5 months inside a laboratory or outside with direct sun exposure.

Daily relative humidity (RH) means ranged from 23.0 ± 0.0 to 35.1 ± 0.5% in woven bags kept outside, and 27.5 ± 0.0 to 38.7 ± 0.0% for those kept inside (Fig. 1a). For PICS bags, daily RH varied from 26.6 ± 0.1 to 41.8 ± 0.3% in bags kept outside and 23.8 ± 0.0 to 31.6 ± 0.0% for those stored inside. There was an increase in the average daily RH in woven bags (1.5–11.0%) stored inside compared to those kept outside for the period from January to April 2016. For the PICS bag, an average increase in daily RH of 1.89–12.4% was observed in bags stored outside compared to those stored inside. The correlations between the average daily RH and treatments are -12.8% (p = .12) and -20.0% (P = .41) for woven and PICS bags, respectively, kept outside; and 9.6% (p = .25) for PICS bags stored inside the laboratory.

The average daily temperature ranged from 21.3 ± 0.2 to 42.4 ± 0.3 °C and 24.9 ± 0.0 to 41.4 ± 0.0 °C in woven bags kept outside and inside, respectively (Fig. 1b). For PICS bags, temperatures varied from 20.0 ± 0.1 to 39.7 ± 0.2 °C and 23.7 ± 0.0 to 36.0 ± 0.0 °C for the bags stored outside and inside, respectively. From mid-January to mid-March, there was an average daily temperature increase of 2.5–18.1 °C in woven bags compared to PICS bag when stored outside. The correlations with the average daily ambient temperature were 60.3% (P < .01) for woven bag kept outside and 71.6% (P < .01) for those stored inside the lab and; 96.9% (P < .01) and 96.1% (P < .01) for PICS kept outside and inside, respectively.

**Fig. 2:**
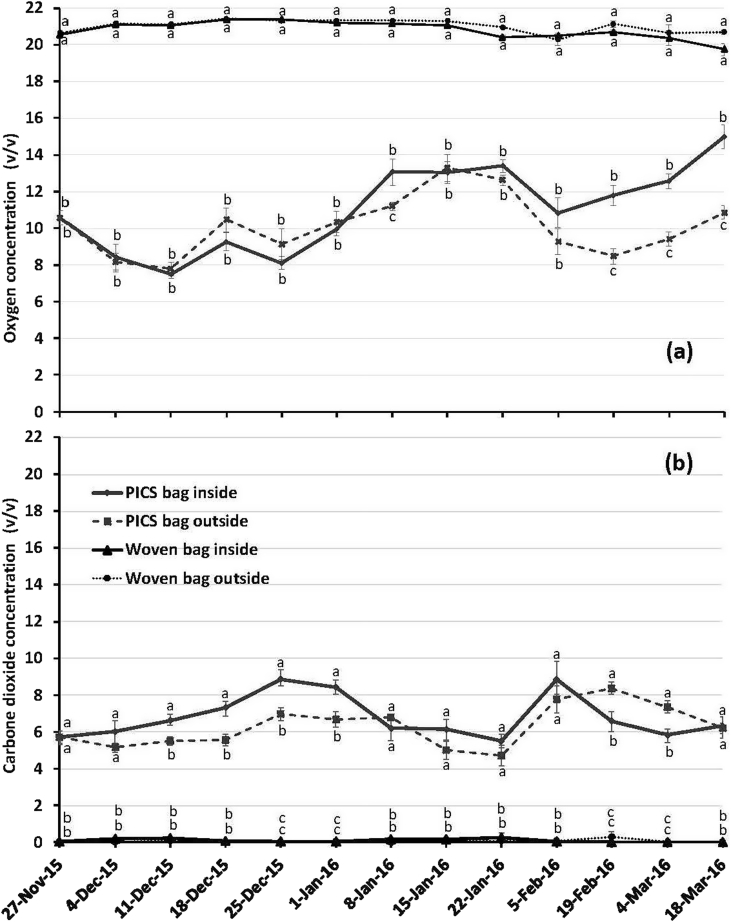
Variation in O_2_ (a) and CO_2_ (b) levels in PICS bags and woven bags filled with naturally infested cowpea and stored at the INRAN Maradi Station in Niger for 4.5 months inside a laboratory and outside with direct sun exposure. Points (S.E.M) associated with the same letter are not significantly different (LSD, 5%). X You need to explain what the Y axis units are in each block. The X-axis labels are of different sizes. They should be the same size. You need to put “A” and “B” labels on the figures.

Throughout the experiments, oxygen levels were lower in PICS bag treatments compared to woven bags (Fig. 2a). On the second day of the experiment (27 November 2015), an average oxygen level of 10.6 ± 0.4% (v/v) was observed in PICS bag treatments and 20.5 ± 0.2 to 20.6 ± 00% (v/v) in woven bags (F = 197.00, P < .01) stored inside and outside the laboratory respectively. At day 114 (18 March 2016), the mean oxygen level in PICS bags varied from 10.9 ± 0.4 to 15.00 ± 0.6% (v/v) and from 19.8 ± 0.3 to 20.7 ± 0.4% (v/v) in woven bag (F = 73.77, P < .01) stored inside and outside of the laboratory. The CO_2_ levels were higher in PICS bags compared to those in woven bags regardless of whether the bags were stored inside or outside the laboratory (Fig. 2b). Two days after bag closure (27 November 2015), CO_2_ levels of 5.7 ± 0.6 to 5.7 ± 0.2% (v/v) were noted in PICS bag treatments while in woven bags the levels were below detection. At that 114^th^ day (18 March 2016), the average level of CO_2_ in PICS bag treatments ranged from 6.2 ± 0.3 to 6.3 ± 0.3% (v/v), whereas it was negligible for woven bag treatments.

Initial infestation levels averaged 5.4 *C. maculatus* adults per 500 g of grain (Table 1). After 4.5 months of storage, we observed high (77.8–88.9%) mortality in PICS bags, while in woven bags, the adult infestation level had increased 3.8 to 4.7 times compared to the initial level. The numbers of larvae, pupae and adults found in the grain and the weight of 100 seeds in the PICS bag treatments after 4.5 months of storage did not differ from what was observed when the experiment was set up (Table 2). By contrast, the numbers of insects in woven bags increased by 2.2–6 times and this was accompanied by a weight loss of 29.2% for bags stored in the laboratory and 16.7% for those stored outside exposed to the sun. The number of emergence holes per 100 cowpea seeds also increased by 73.2% in woven bags stored inside the laboratory and 88.5% for those kept outside. Grain held in PICS bag had no more emergence holes after 4.5 months of storage that it did at the outset of the experiment. After 4.5 months of storage, germination rates of grain held in PICS bag stored in the laboratory was similar to that noted at the beginning of the experiment (Table 3). By comparison, there was an average reduction in germination rate of 7.6% for PICS bag stored outside; 16.1% for woven bags stored in the laboratory, and 60.0% for woven bags stored outside.

**Table 1:** Adult bruchids found in cowpea grains naturally infested and stored in PICS bags and woven polypropylene bags at the INRAN Maradi Station in Niger for 4.5 months inside a laboratory or outside with direct sun exposure. Means followed by the same letter are not significantly different (LSD. 5%).

Treatments	n	Living adults of *C. maculatus* in 500 g of cowpea grains
Initial infestation	64	5.4 ± 0.5**a**
PICS inside after 4.5 months	18	1.2 ± 0.3**a**
PICS bags outside after 4.5 months	18	0.6 ± 0.3**a**
Woven bag inside after 4.5 months	18	25.6 ± 6.3**b**
Woven bag outside after 4.5 months	18	20.6 ± 7.5**b**
**ANOVA**		**F**=**17.05; P**<**.01**

**Table 2:** Infestation and damage to cowpea grains naturally infested and stored in PICS bags and woven bags at the INRAN Maradi Station in Niger for 4.5 months inside a laboratory and outside with direct sun exposure. Means followed by the same letter are not significantly different (LSD. 5%).

Treatment	n	*C. maculatus* infestation per 100 seed			*C. maculatus* damage	
		Pupae	Alive larvae	Adults	100 grains weight	% of grains with holes
Initial infestation and damage	108	1.6 ± 0.1**a**	0.9 ± 0.1**a**	3.2 ± 0.2**a**	14.4 ± 0.0**a**	11.5 ± 0.4**a**
PICS inside after 4.5 months	54	1.8 ± 0.1**a**	0.4 ± 0.1**a**	3.5 ± 0.2**a**	14.8 ± 0.1**a**	11.6 ± 0.4**a**
PICS bags outside after 4.5 months	54	1.8 ± 0.2**a**	0.6 ± 0.1**a**	3.5 ± 0.3**a**	14.6 ± 0.2**a**	12.0 ± 0.5**a**
Woven bag inside after 4.5 months	54	3.5 ± 0.6**b**	3.1 ± 0.5**c**	19.6 ± 1.9**b**	10.2 ± 0.2**c**	84.7 ± 2.9**b**
Woven bag outside after 4.5 months	54	6.7 ± 0.5**c**	2.4 ± 0.3**b**	18.4 ± 0.9**b**	12.0 ± 0.1**b**	100.0 ± 0.0**c**
**ANOVA**		**F**=**56.25; P**<**.01**	**F**=**22.60; P**<**.01**	**F**=**180.05; P**<**.01**	**F**=**130.41; P**<**.01**	**F**=**1878.00; P**<**.01**

**Table 3:** Germination rate of cowpea grains naturally infested and stored in PICS bags and woven bags at the INRAN Maradi Station in Niger for 4.5 months inside a laboratory and outside with direct sun exposure. Means followed by the same letter are not significantly different (LSD. 5%).

Treatments	n	Germination rate (%)
Initial germination rate	72	70.4 ± 2.1**a**
PICS inside after 4.5 months	36	74.3 ± 1.9**a**
PICS bags outside after 4.5 months	36	62.8 ± 1.9**b**
Woven bag inside after 4.5 months	36	54.3 ± 3.6**c**
Woven bag outside after 4.5 months	36	10.4 ± 1.5**d**
**ANOVA**		**F**=**76.54; P**<**.01**

PICS bags and woven bags exposed to the sun suffered damage (Table 4). Visible degradation of woven bags stored outside became apparent between 31 and 49 days after beginning the experiment; with an average of 39.3 ± 7.6 days. For PICS bags exposed to sun, the first obvious degradation of the outer woven bags was noticed between 29 and 41 days. The PICS woven bags maintained their integrity for an average of 36.2 ± 4.6 days. The middle liner became damaged between 90 and 137days, while inner liner between 123 and 145 days. The damage to the middle liner was noted after an average of 110 ± 8.33 days, while that of the inner liner after 135 ± 3.99 days. The inner polyethylene PICS liners took longer before they showed evident damage. The average breaking time of the two liners was 122 days. No damage was noted on PICS and woven bags stored in the laboratory.

**Table 4:** Number of days before damage was apparent in each of the three PICS bag layers and in control woven bags stored outside with direct sun exposure for 4.5 months at the INRAN Maradi Station in Niger.

Number of days (d) before damage				
Replication	PICS bag			Control
	Woven	Middle HDPE Liner	Inner HDPE Liner	Woven
Bag 1	41	91	143	46
Bag 2	35	90	138	39
Bag 3	29	94	139	28
Bag 4	41	123	123	43
Bag 5	39	123	123	49
Bag 6	32	137	145	31
**Mean**	**36 **±** 2.04**	**110 **±** 8.33**	**135 **±** 3.99**	**39 **±** 3.41**

## 4 Discussion

Our observations have assessed the performance of PICS bags in maintaining grain quality under the extreme environmental conditions obtained in the Sahelian zone of Africa. PICS bags stored outside with daily direct exposure to the sun for 10 or 11 h can still preserve cowpea grain for up to 4.5 months in a Sahelian environment under average daily temperatures ranging from 11.4 to 44.6 °C. Despite the extreme conditions, the PICS bags still maintain airtight conditions that cause high mortality and arrest population growth of *C. maculatus*. PICS bag exposed to the sun were as effective as those stored inside the laboratory. Our present results confirm the findings of previous studies showing that PICS bags preserve cowpea grain extremely well (Baoua et al., 2012, Baoua et al., 2015, Murdock and Baoua, 2014) when they are stored inside buildings. Substantial protection for some months even occurs when the bags are exposed to sun and weather.

Average daily temperatures in PICS bags held outside or inside the laboratory remained highly correlated and dependent on the prevailing ambient temperature. Williams et al. (2017) reported similar results after testing PICS technology for maize storage in the USA. As regards relative humidity, there was no significant correlation between RH values recorded within in PICS bags and outer RH when they were stored inside or outside the laboratory. Despite high daytime and low nighttime temperatures, PICS bags buffer cowpea grain from variations of ambient humidity, which is in agreement with results reported by Baoua et al. (2014) with maize stored in PICS bags. Relative humidity in PICS bags stored inside and outside the laboratory remained relatively constant with variations of less than 10% for the 4.5 months of storage. During this same period ambient RH varied more than 30%. PICS bags stored outside had relatively higher internal RH compared to bags stored inside the laboratory and the gap increased with time. The effect of continued exposure to the sunlight and varying internal temperatures combined with slow deterioration of the bags over time probably account for the higher RH in PICS bags stored outside.

Degradation of the polypropylene outer woven bag was likely due to the effect of sunlight and high temperatures on the organic chemical compounds that make up the plastic bags material. Radiant energy leads to a reorganization of the polymer chains which affects the flexibility and structure of the plastic (Tocchetto et al., 2001, Khan and Ahmed, 2003). We expected that the PICS woven bag exposed to the sun would deteriorate given that they are not UV-treated to protect them against the sun's rays. This is not typically a problem because farmers store their grain-filled bags inside their compounds. We found that liners are less affected by sunlight than the polypropylene outer bag. This may be explained by the sturdiness of the liner bag and the higher plastic density, which is 1.2 times higher than that of the woven bag. These two factors slowed the degradation of the inner two liners by higher temperatures and solar radiation. An important contributing factor was that the inner polyethylene liners were initially shielded from the sunlight by the outer woven polypropylene bags. The presence of two liners in each PICS bag contributed to the performance of PICS bags under these harsh environmental conditions. Exposure of PICS bags to the sun caused progressive deterioration first of the woven outer bag and then, in sequence, the middle and inner liners. Obviously, such serious damage limit the reuse of the bags for grain storage. We have shown earlier that PICS bags stored inside are reused by farmers for 3 storage seasons on average (Baributsa et al., 2014, Baoua et al., 2013).

Exposure of PICS bags to the sun affected the cowpea grain stored outside by decreasing germination by 7.6% compared to the germination rate at the outset of storage. This modest reduction in germination probably result from the swings in relative humidity resulting from the condensation and evaporation as well as the large variations in daily temperatures. Germination of grain held in woven bags was severely affected, most of it primarily because of damage to the grain caused by insects developing in these bags. There was a still greater drop in germination of grain stored in woven bags outside -- a decrease of 60% compared to the initial germination and at least 5 times less than that seen in grain in woven bags stored inside. Increases in temperature in woven bags stored outside due to the extremely high exterior temperatures as well as to bruchid activity resulted in an increase in the proportion of grains with holes (73.2% inside to 88.5% outside) and seed weight losses (16.6%–29.2%). Damage to grain measured as emergence holes and grain weight loss appeared to be slightly higher in woven bags stored outside than those kept inside. Higher temperatures in bags exposed to sunlight may have contributed to greater insect feeding and reproductive activity and hence increasing damage to cowpea grains stored outside. We noted that variations in temperatures and RH resulted in seed cracking and contamination by other microorganisms, etc. but we did not investigate these parameters further.

This study gave us new information on the performance of PICS bag when exposed to extreme environmental conditions of the Sahel. Despite the harsh conditions, PICS bags stored outside can maintain grain quality and good germination rates for several months. Clearly it is strongly preferable to store PICS bags inside, where they are exposed to milder ambient conditions and are not exposed to sunlight. But in harvest situations and in the difficult world of international development or aid emergencies where large shipments of grain may occur and arrive in localities where there is limited storage space, PICS bags could be held outside for a limited time, if shielded from livestock and thieves, and be safely used to store grain and prevent losses to bruchids. Once interior storage space became available, they could be moved inside to more favorable conditions. Exposure to the sun should be avoided if at all possible. The performance of the PICS bags for a few months outside is minimally affected by exposure to the sun, but the longevity and reuse of the bags are reduced.
